# Systemic steroids for cervical radicular pain: A systematic review

**DOI:** 10.1016/j.inpm.2023.100280

**Published:** 2023-10-01

**Authors:** Nolan Gall, Cyrus Ghaffari, Jyotsna Koduri, Chris Dove, Joshua Levin

**Affiliations:** a Department of Anesthesiology and Critical Care, Division of Pain Medicine, Philadelphia, PA, USA; b Department of Orthopaedic Surgery, Division of PM&R, Stanford University, Redwood City, CA, USA; c Department of Neurosurgery, Stanford University, Palo Alto, CA, USA

**Keywords:** Oral steroids, Intravenous steroids, Intramuscular steroids, Cervical radiculopathy, Cervical radicular pain

## Abstract

**Objective:**

Determine the effectiveness of systemic steroids for the treatment of cervical radicular pain.

**Data sources:**

We identified articles from two electronic databases (PubMed and Ovid Medline) and previously known articles.

**Study selection:**

We combined (using the Boolean Operator “AND”) the following search terms: “Steroid* and Cervical Radic*” or “Steroid* and Cervicobrachialgia”. We applied the search to PubMed and Ovid Medline research databases for all studies up February 11, 2022. We included all published articles involving cervical radicular pain treated with systemic steroids among adult users (≥18 years old). We defined systemic steroids as steroid medication administered into the body excluding epidural or intra-articular injections. We excluded reviews and editorials.

**Data extraction:**

Information extracted from each study included: (1) study design; (2) characteristics of trial participants; (3) diagnostic criteria; (4) treatment intervention protocol; (5) outcome measure; and (6) follow-up time. Outcomes included the neck disability index (NDI) and patient reported pain.

**Data synthesis:**

842 publications were identified, 7 were suitable for inclusion. The only level one study comparing oral steroid to placebo reported greater improvements of NDI (35.7 ± 21.4 versus 12.9 ± 10.2, P < 0.001) and NPRS (4.4 ± 2.7 versus 1.6 ± 1.2, P < 0.001), and higher success rates (76% [95% CI: 60–92%] vs 30% [95% CI: 14–46%]) in the oral steroid group compared to the placebo group. The only level one study comparing paraspinal intramuscular (IM) versus interlaminar (IL) epidural steroids reported worse outcomes in the IM group with a success rate of 35% [95% CI: 13–58%] at 1 week and 12% [95% CI: 0–27%] at one year in the IM group, compared to 76% [95% CI: 60–92%] at 1 week (p = 0.04) and 68% [95% CI: 50–86%] at one year (p = 0.0004) in the IL group (P < 0.0004). The Cochrane Risk of Bias Tool and GRADE system was used to assess bias and rate the overall evidence quality.

**Conclusions:**

Very limited evidence exists supporting treatment of cervical radicular pain with systemic steroids. Oral steroids can be considered as a reasonable conservative option based on one RCT, whereas IM steroids may be inferior to epidural steroids based on another RCT. Additional higher quality studies are needed.

## 1 Introduction

Cervical radiculopathy is a common disabling disease with an annual incidence of 83 per 100,000 people [1]. Fortunately, the favorable natural history of the disease results in only approximately 26% of patients electing for operative management [1], leaving conservative management of pain and disability as the foundation of treatment.

Systemic steroids are commonly used treatments that have been studied extensively for lumbar radicular pain, with overall negative results [2]. However, very little evidence exists for the use of systemic steroids for cervical radicular pain. On the basis of anecdotal experience, physicians often use oral steroid tapers as a pharmacologic treatment for cervical radicular pain [3] prior to considering cervical epidural steroid injections due to the risk of catastrophic complications from these injections [4,5]. With evidence showing limited benefit from cervical epidural steroid injections (ESIs) [6,7], as well as the risks associated with such procedures, management with systemic steroids is worthy of investigation.

In order to try to improve the quality of conservative spine care and better evaluate the effectiveness of systemic steroids for the treatment of cervical radicular pain, the purpose of this review is to A) compile the current literature on systemic steroids in the treatment of cervical radicular pain and B) analyze and grade its effectiveness.

## 2 Methods

We conducted this study according to the Preferred Reporting Items for Systematic Reviews and Meta-Analysis (PRISMA) statement [8]. This IRB-exempt study was registered on PROSPERO.

## 3 Search strategy

We identified studies by searching two electronic databases (PubMed and Ovid Medline). We combined (using the Boolean Operator “AND”) the following search terms: “Steroid* and Cervical Radic*” or “Steroid* and Cervicobrachialgia”. We applied the search to PubMed and Ovid Medline research databases for all studies up to the present date. The last search was run on February 11, 2022. Two reviewers (NG, CG) performed eligibility assessment independently in a standardized manner. Disagreements between reviews were resolved by consensus. One author was attempted to be contacted for clarifying information regarding mean follow up time but no response was received [9]. Bias was assessed utilizing the Cochrane Risk of Bias Tool [10] and overall evidence quality was rated by the Grading of Recommendations Assessment, Development and Evaluation [11].

## 4 Inclusion/exclusion criteria

We included all published articles involving cervical radicular pain treated with systemic steroids among adult users (≥18 years old). We defined systemic steroids as steroid medication administered into the body excluding epidural or intra-articular injections. Information extracted from each study included: (1) study design (2) characteristics of trial participants (3) diagnostic criteria; (4) treatment intervention protocol; (5) outcome measure; and (6) follow-up time. We excluded reviews and editorials.

## 5 Statistical analysis

Outcomes measures were extracted across all studies. Differences were evaluated across all included studies in addition to secondary outcomes, as defined by the studies. 95% Confidence Intervals (CI) were calculated for two studies [9,12]. We defined statistical significance as alpha less than 0.05.

## 6 Results

Reviewers achieved 100% agreement on the papers selected for inclusion in this article.

**Fig. 1:**
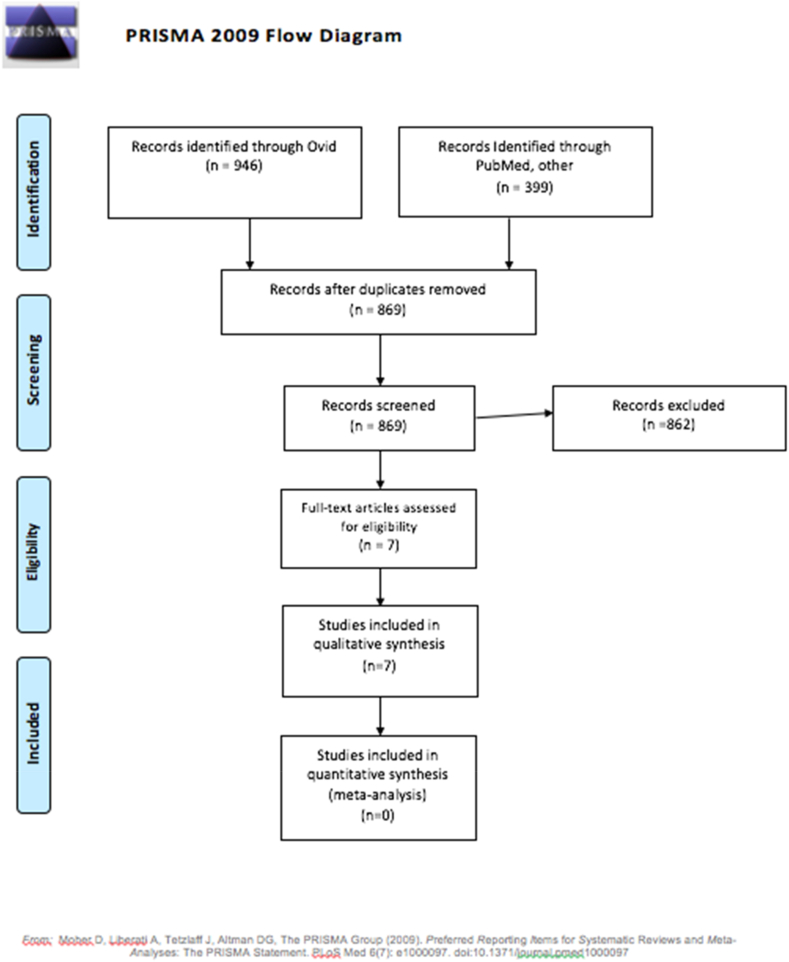
Systematic review flow diagram.

We identified a total of 1345 articles (Fig. 1), of which 476 were duplicates and an additional 862 articles were excluded after reading the title and abstract.

The characteristics of the studies included in this review are listed in Table 1, oral steroids were used as the treatment in six studies [9,[13], [14], [15], [16], [17]], while intramuscular steroids (IM) were used in one study as a comparison to interlaminar (IL) epidural steroid injections [12]. No studies used intravenous steroids. Two studies were randomized controlled trials (RCT) [9,12], two were prospective cohort studies [13,14], two were case reports [15,17], and one was a retrospective chart review [16]. We considered one of the prospective cohort studies as a retrospective study for our purposes because the researchers retrospectively assessed patients’ pain response to an oral steroid treatment course for their prospective study [13]. Two studies utilized oral methylprednisolone tapers [15,17], two studies utilized oral prednisone tapers [9,14], and two studies did not report the type of oral steroid that was utilized [13,16]. One study utilized IM methylprednisolone [12]. Four studies recorded pain via a patient reported pain scale [9,12,16,17], and one reported a functional outcome measure [9]. Four studies reported a follow up time frame [12,14,16,17].

**Table 1:** Study characteristics.

Author, Year	Study Design	Population (Mean age, SD)	Diagnosis	Intervention	Control	Primary Outcome	Follow-up Time
Stav, 1993 [12]	Randomized, controlled trial	N = 17 (49.3, 3.00)	Cervicobrachialgia for at least 6 months had clinical and radiological signs of pathology in the C4–C7 region, with or without signs of mechanical pressure on the dura mater and/or the nerve root.	Interlaminar epidural injection of 80 mg (2 ml) of methylprednisolone sodium acetate and 5 ml 1% lidocaine	Paraspinal intramuscular injection of 80 mg (2 ml) of methylprednisolone sodium acetate and 5 ml 1% lidocaine	Patient reported visual analog scale	1 week and 1 year
Saal, 1996 [14]	Longitudinal Cohort Study	N = 28 (43.1, 2.7)	Focal cervical disc protrusion of less than 4 mm identified on MRI and a chief complaint of upper extremity pain compatible with cervical radiculopathy	Oral prednisone (max 60 mg/day ×3 days) taper over 1 week	None	Patient reports adequate symptom control	2.3 ± 0.3 years (Mean)
Stitik, 1999 [15]	Case Report	N = 2 (57, 0)	Radicular pain and electromyography	Oral methylprednisolone 24 mg/day taper over 6 days	None	Patient reported pain	NR
Nortman, 2006 [16]	Retrospective Chart Review (Abstract)	N = 21^b^	Signs and symptoms of radiculopathy	Oral Steroids	None	Numerical Pain Rating Scale (0–10)	Within 6 weeks
Mitra, 2008 [17]	Case Series	N = 1 (38)	Myotomal pain and weakness with MR imaging confirmation of perineural cyst at C6 nerve root	Oral methylprednisolone 24 mg/day taper over 6 days	None	Patient reported pain via visual analog scale and physician manual motor testing	3 months
Ghasemi, 2013 [9]	Randomized, controlled trial	N = 59 (46.2, 9.0)	Neck or shoulder pain and confirmed by electromyography and MR imaging of cervical spine.	Oral prednisone 50 mg/day taper over 5 days	Placebo	Neck Disability Index, Numerical Pain Rating Scale (0–10)	NR
Crovo, 2017 [13]	Retrospective Study^a^	N = 71 (51.8, 11.0)	Acute cervical radicular pain with no clinically significant weakness of at least six weeks' duration and had undergone magnetic resonance imaging of the cervical spine in the past six months.	Oral Steroids	None	Any report of greater than 0% pain relief during the course of oral steroid treatment expressed as a binary variable (yes/no).	NR

Not reported (NR).

a Original study consisted of a prospective cohort study that retrospectively assessed data of interest to this study.

b Total N = 100 (38.9 ± 9.2) which included both lumbar (79) and cervical radiculopathy (21).

Study interventions and reported outcome data, including both quantitative and qualitative, were organized and included in Table 2.

**Table 2:** Study results.

Author, Year	Intervention	Mean Pre-Intervention Outcome (SD)	Mean Post-Intervention Outcome (SD)
Stav, 1993 [12]	Interlaminar epidural versus paraspinal intramuscular injection of 80 mg (2 ml) of methylprednisolone sodium acetate and 5 ml 1% lidocaine	No baseline pain data reported	One week after the last injection, the IL group experienced better relief with 76% [95%CI: 60–92%] of patients achieving at least 50% pain relief compared to 35% [95%CI: 13–58%] in the IM group (P < 0.0377). At the one year follow up the IL group experienced similar results with 68% [95% CI: 50–86%] achieving at least 50% pain relief compared to 12% [95%CI: 0–27%] in the IM group (P < 0.0004).
Saal, 1996 [14]	Oral prednisone (max 60 mg/day ×3 days) taper over 1 week	Radicular pain	13/22 received “adequate control” of symptoms
Stitik, 1999 [15]	Oral methylprednisolone 24 mg/day taper over 6 days	Radicular pain	“Symptoms resolved"
Nortman, 2006 [16]	Oral Steroids	No Cervical Specific data‡	No Cervical Specific data‡
Mitra, 2008 [17]	Oral methylprednisolone 24 mg/day taper over 6 days	VAS: 9/10	VAS: 6/10
		Wrist extension MMT: 4/5	Wrist extension MMT: 5/5
Ghasemi, 2013 [9]	Prednisone 50 mg/day taper over 5 days	NPRS: 8.4 (1.5)	NPRS: 4.0 (2.6)*
		NDI: 68.8 (17.5)	NDI: 33.1 (24)*
Crovo, 2017 [13]	Oral Steroids	N = 70 with radicular pain	N = 49 with >0% pain improvement

*Statically significant (P < 0.05).

‡Pretreatment and post treatment pain scores for both lumbar and cervical patients were 7.35 ± 2.46 and 5.27 ± 2.91, respectively (mean difference 2.09 ± 2.76; 95% CI: 1.54, 2.63; P < 0.001).

NG and JL assessed risk of bias of included RCTs using the Cochrane Risk of Bias Tool [10]. The Cochrane Risk of Bias Tool is structured into a fixed set of domains of bias, focusing on different aspects of trial design, conduct, and reporting. It is based on signaling questions that algorithmically result in different categories of “risk” for bias. Discrepancies were resolved by consensus discussion in order to reach a final decision.

### 6.1 Randomized controlled trials

In 1993, Stav et al. published results of a single center, non-blinded, randomized controlled trial evaluating the efficacy of IL epidural steroid injections versus paraspinal IM steroid injections for refractory cervicobrachialgia [12]. The patients all had clinical and radiological signs of pathology in the C4–C7 region. Patients were randomized into treatment arms for an IL epidural injection or a paraspinal IM injection of 80 mg (2 ml) of methylprednisolone sodium acetate and 5 ml of 1% lidocaine. Patients received between 1 and 3 injections. Pain relief was measured by visual analog scale (VAS) at one week and one-year post-treatment by a blinded physician. There was no significant difference in patient population or mean number of injections between groups. One week after the last injection, the IL group experienced better relief with 76% [95%CI: 60–92%] of patients achieving at least 50% pain relief compared to 35% [95%CI: 13–58%] in the IM group (P < 0.0377). At the one-year follow up, the IL group experienced similar results with 68% [95% CI: 50–86%] achieving at least 50% pain relief compared to 12% [95%CI: 0–27%] in the IM group (P < 0.0004). No procedural complications occurred, and no adverse events were recorded. Of note, 8 of the original 25 patients in the IM steroid group were excluded from the analysis because they “started the process of litigation of insurance claims, and they were excluded from the study, since their subjective analysis of pain relief might not be reliable.” None of the 25 patients in the IL ESI group were excluded.

In 2013, Ghasemi et al. published results of a single center, double-blinded randomized placebo-controlled trial evaluating the efficacy of oral steroids for treating acute cervical radicular pain^9^**.** This study population included adult patients with neck or shoulder pain for less than one month and a Neck Disability Index (NDI) score of 15 or more (indicating moderate disability). All patients underwent electrodiagnostic testing and magnetic resonance imaging (MRI) of the cervical spine. The pre-intervention (prednisone 50 mg/day taper over 5 days, 325 mg of acetaminophen three times a day, and ranitidine 150 mg two times per day) numeric pain rating scale (NPRS) and NDI were homogenous across both study groups. Both study groups received acetaminophen and ranitidine in addition to either the steroid or placebo. Following intervention, the prednisone group experienced greater reductions in NDI (35.7 ± 21.4 versus 12.9 ± 10.2, P < 0.001) and NPRS (4.4 ± 2.7 versus 1.6 ± 1.2, P < 0.001) as compared with the placebo group. Based on the minimal clinically important change in NDI (NDI = 8.5), pain was improved in 76% (22/29) [95%CI: 60–92%] of the prednisolone group and 30% (9/30) [95%CI: 14–46%] of the placebo group (P < 0.001). No follow up time frame was reported, nor were patients tracked for recurrence or progression to epidural injections or surgery. Our attempt to contact the authors to inquire about the follow up time frame was unsuccessful.

### 6.2 Retrospective study

In 2017, Crovo et al. published a single center prospective study evaluating if pain reduction following oral steroid treatment predicted pain reduction after an interlaminar cervical epidural steroid injection (ESI) [13]. This study included patients with acute radicular pain of at least six weeks duration who were referred for a cervical ESI. On the day of the first injection, patients completed a retrospective assessment of their greatest relief obtained from oral steroids, expressed as 0–100%, and how many days of relief were obtained. Success from prior treatment with oral steroids was defined categorically as “any report of greater than 0% in pain relief during the course of oral steroid treatment expressed as a binary variable”. Forty-nine patients (69% [95%CI: 58%–80%]) reported success whereas 22 (31% [95%CI: 20–42%]) reported failure. No data was reported on the degree of pain relief. There was no significant difference in the numerical rating scale (NRS) scores at six months post-ESI between those who reported success from the oral steroids and those who did not (P = 0.88). The researchers concluded that patients can be reassured that they may experience pain reduction after a cervical epidural steroid injection despite failure of oral steroid therapy.

### 6.3 Longitudinal cohort study

In 1996, Saal et al. published a study evaluating a systematically and uniformly applied treatment program with increasing intervention as further pain control was needed [14]. This study included patients with a chief complaint of upper limb pain consistent with cervical radicular pain from a focal cervical disc protrusion of less than 4 mm. All patients began treatment with ice, relative rest, a hard cervical collar worn for up to 2 weeks in a position to maximize arm pain reduction, non-steroid anti-inflammatories for 6–12 weeks, manual and mechanical traction in physical therapy, and progressive strengthening exercises of the shoulder girdle and chest with training in postural control and body mechanics. A total of 28 patients were enrolled with 4 patients lost to follow up. Of the remaining 24 patients, 22 did not respond to the aforementioned treatment regimen, and were then treated with a single week course of oral prednisone 60 mg per day for 3 days, followed by a rapid taper. 13/22 (59% [95%CI: 38–80%]) subjects achieved self-defined “adequate pain control” of symptoms, and the remaining 9 patients (41%) progressed to a cervical ESI. When evaluating the effect of their systematically and uniformly applied treatment program, 20/24 (83% [95%CI: 68–98%]) subjects had a good (minimal limitations in activity level, minor pain complaints from neck pain only, fully satisfied with outcome) or excellent outcome (no limitations in activities, no pain, fully satisfied with outcome).

### 6.4 Retrospective chart review

In 2006, Nortman et al. published an abstract retrospectively evaluating the use and effectiveness of oral corticosteroids for acute radicular pain [16]. This study included both cervical and lumbar patients who were treated with oral steroids (unknown type or dose) within 3 months of symptom onset and had documentation of pre- and post-treatment NPRS (0–10) scores. Of the 100 patients included, 21 patients had cervical radicular symptoms, with 11/21 (52%) having confirmatory evidence of radiculopathy (defined as MRI, EMG, or objective clinical findings). After treatment, NPRS scores improved from 7.4 ± 2.5 to 5.3 ± 2.9 (mean difference 2.1 ± 2.8; 95% CI: 1.5, 2.6; P < 0.001), however scores were not stratified by lumbar or cervical location, so the success for patients with cervical radicular symptoms is unknown. None of the independent factors in the regression analysis model were significant predictors of pain reduction.

### 6.5 Case reports

In 1999, Stitik et al. reported on two patients with cervical radicular pain after manipulation via a salon sink while having their hair washed [15]. Both patients had an EMG confirmed radiculopathy (one at C8/T1 and one at C7). Both patients were treated with a methylprednisolone 24 mg/day taper over 6 days, and both reported that their “symptoms resolved”.

In 2008, Mitra et al. reported on two patients with radicular pain presumed to be caused by a perineural cyst [17]. One patient presented with cervical radicular pain and one with lumbar radicular pain. The patient with cervical radicular pain presented with dermatomal pain, 4/5 wrist extensor weakness, and a perineural cyst adjacent to the C6 nerve root. The patient was treated with an oral methylprednisolone 24 mg/day taper over 6 days. At the 3-month follow-up, the VAS improved from 9/10 to 6/10, and the wrist extensor weakness had resolved.

## 7 Tolerance of systemic steroids

Adverse event data was not collected by one of the studies. Of the remaining six, no adverse events were reported (Table 3).

**Table 3:** Adverse events.

Author, Year	Intervention	Adverse Events
Stav, 1993 [12]	Intramuscular methylprednisolone	None Reported
	80 mg and 5 ml of 1% lidocaine	
Saal, 1996 [14]	Oral prednisone (max 60 mg/day ×3 days) taper over 1 week	None reported
Stitik, 1999 [15]	Oral methylprednisolone 24 mg/day taper over 6 days	None reported
Nortman, 2006 [16]	Oral steroids	None reported
Mitra, 2008 [17]	Oral methylprednisolone 24 mg/day taper over 6 days	None reported
Ghasemi, 2013 [9]	Oral prednisone 50 mg/day taper over 5 days	Not assessed
Crovo, 2017 [13]	Oral steroids	None reported

## 8 Synthesis of results

The reviewed studies were found to be heterogeneous across trial type (only one placebo-controlled RCT), interventions (different steroid types/doses/administrations), and outcome measurements. For these reasons, meta-analysis of comparative measures of effect was not performed.

## 9 Quality of evidence

The grading of recommendations assessment, development and evaluation (GRADE) system was used to rate the overall quality of evidence of the RCTs included in our study [11]. Ghasemi et al.‘s trial comparing oral steroids with placebo had an overall low risk of bias [9] (Table 4). According to the GRADE system, due to trial limitations regarding lack of follow up time frame, the GRADE classification was downgraded to moderate quality evidence that oral steroids are superior to placebo for the treatment of cervical radicular pain. Stav et al.‘s unblinded RCT comparing IM steroid with ESI had an overall moderate risk of bias (Table 4) given the lack of blinding and exclusion of patients exclusively from the IM steroid group who started a litigation process after randomization [12]. Due to trial limitations aforementioned, the GRADE classification was downgraded to low quality evidence that IM steroids are inferior to ESI.

**Table 4:** Cochrane risk of bias assessment.

Author, Year	Study Comparison	Bias Related to Random-ization Process	Bias Related to Deviations from Intended Interventions	Bias Related to Missing Outcome Data	Bias Related to Outcome Measurement	Bias Related to Selection of Reported Results	RCT Other Bias Source	RCT Overall Risk of Bias
Stav et al., 1993 [12]	Interlaminar epidural versus paraspinal intramuscular injection of 80 mg (2 ml) of methyl-prednisolone sodium acetate and 5 ml 1% lidocaine	Low risk	Low Risk	Low risk	No reported baseline pain scores for either group	No reported sensory or motor deficits data reported but mentioned no significant improvements in the text.	Exclusion of 8 patients after randomization, exclusively from the IM steroid group, because they started the process of litigation; No reported blinding.	Moderate Risk
Ghasemi et al., 2013 [9]	Prednisone 50 mg/day taper over 5 days	Low risk	Low risk	Low risk	Low risk	No follow up time frame reported	None identified	Low risk

## 10 Discussion

There are few studies that assess the use of systemic steroids for the treatment of cervical radicular pain. The majority of the studies published were non-randomized studies including case reports and cohort studies. The only placebo-controlled RCT, conducted by Ghasemi et al., reported greater improvements in NDI (35.7 ± 21.4 versus 12.9 ± 10.2, P < 0.001) and NPRS (4.4 ± 2.7 versus 1.6 ± 1.2, P < 0.001) in the oral steroid group compared to placebo. While there was a fair bit of overlap in these results, the differences were significant. Additionally, categorical data was presented demonstrating better success rates based on the minimal clinically important change in NDI (NDI = 8.5), with 76% (22/29) [95%CI: 60–92%] of the prednisolone group achieving success compared to 30% (9/30) [95%CI: 14–46%] of the placebo group (P < 0.001) [9]. Stav et al. conducted the only invasive RCT included in our review, comparing IM steroid injections with IL epidural steroid injections [12]. In this study, the ESI group outperformed the IM group, however the lack of blinding and the excluded data exclusively from one of the treatment arms (the IM steroid group) limit the conclusions that can be drawn.

Using the GRADE classification [11], we found moderate quality evidence that oral steroids are superior to placebo for the treatment of cervical radicular pain, however this conclusion is based on only one study. Also, the follow-up time frame in this study was not reported, so the duration of effect is unknown. For IM steroids, we found low quality evidence that IM steroids are inferior to ESIs. There were no studies evaluating IM steroids versus placebo.

While studies evaluating the effectiveness of systemic steroids for the treatment of cervical radicular pain are few, several studies have reported outcomes from systemic steroids for the treatment of lumbar radicular pain, and the results are overwhelmingly negative. A 2011 systematic review by Roncoroni et al. evaluated 7 prospective, double-blind, placebo-controlled RCTs on systemic steroids for the treatment of lumbar radicular pain [2]. They concluded that systemic steroids are not superior to placebo and are associated with more adverse effects (13.3% in steroid group vs 6.6% in placebo group, P < 0.03). Since the publication of this review article in 2011, Goldberg et al. published a prospective, double-blind, placebo-controlled RCT demonstrating a small, yet statistically significant, improvement in function in both the short and long term, but no improvement in lower limb pain in patients with lumbar radicular pain who were treated with oral steroids. The steroid group also reported significantly more adverse effects at three weeks compared to the control group [18].

When patients present with radicular pain, clinicians have several treatment options from which to choose. In the lumbar spine, the literature is clear that systemic steroids are not effective [2]. However, there is strong evidence supporting the use of epidural steroid injections for the treatment of lumbar radicular pain [19]. Therefore, the use of systemic steroids instead of epidural steroid injections for the treatment of lumbar radicular pain is not evidence-based. In the cervical spine, while the literature is less robust, the findings are somewhat conflicting. Anderberg et al. published a prospective RCT demonstrating lack of benefit from cervical transforaminal epidural steroid injections for the treatment of cervical radicular pain [20]. Yet other studies have shown some benefit, with approximately 50% of patients achieving at least 50% relief in pain following cervical epidural steroid injections [6,7], and inferiority of IM compared to epidural steroids [12]. Given that the data supporting the use of cervical ESIs is limited, the risk of catastrophic complications from these injections is small but not zero, and that there is limited data supporting the use of oral steroids in the treatment of cervical radicular pain, the potential implications of the study by Ghasemi et al. are that the use of oral steroids for the treatment of cervical radicular pain appears to be a reasonable option [9]. However, additional high quality studies are needed to further support this conclusion.

## 11 Conclusion

Our systematic review demonstrated that few studies have been published to evaluate the effectiveness of systemic steroids in the treatment of cervical radicular pain. Based on one moderate-quality study, oral steroids are more effective than placebo. Based on one low quality study, intramuscular steroids are less effective than epidural steroid injections. Additional high-quality studies are warranted to further evaluate the safety and effectiveness of systemic steroids as a treatment for cervical radicular pain.

## Funding

The authors have no sources of funding to declare for this manuscript.

## Data availability

The authors confirm that the data supporting the findings of this study are available within the article and its supplementary materials.

## Funding source

None.

## Competing interests

None.

## Conflicts of interest

The authors declare no conflicts of interest.

## Appendix A Supplementary data

PRISMA_2020_checklist.docx PRISMA_2020_checklist.docx
